# The Increased Levels of Fecal Calprotectin in Children With Active Enthesitis Related Arthritis and MRI Signs of Sacroiliitis: The Results of a Single Center Cross-Sectional Exploratory Study in Juvenile Idiopathic Arthritis Patients

**DOI:** 10.3389/fmed.2021.650619

**Published:** 2021-03-08

**Authors:** Lovro Lamot, Marijana Miler, Rudolf Vukojević, Mandica Vidović, Mirta Lamot, Ivana Trutin, Nora Nikolac Gabaj, Miroslav Harjaček

**Affiliations:** ^1^Division of Clinical Immunology and Rheumatology, Department of Pediatrics, Sestre Milosrdnice University Hospital Center, Zagreb, Croatia; ^2^Department of Pediatrics, University of Zagreb School of Medicine, Zagreb, Croatia; ^3^Universirty Department of Chemistry, Sestre Milosrdnice University Hospital Center, Zagreb, Croatia; ^4^Department of Diagnostic and Interventional Radiology, Sestre Milosrdnice University Hospital Center, University of Zagreb, Zagreb, Croatia; ^5^Division of Neonatology, Department of Gynecology and Obstetrics, Sestre Milosrdnice University Hospital Center, Zagreb, Croatia; ^6^Division of Nephrology and Cardiology, Department of Pediatrics, Sestre Milosrdnice University Hospital Center, Zagreb, Croatia

**Keywords:** juvenile idiopathic arthritis, enthesitis related arthritis, juvenile spondyloarthritis, fecal calprotectin, magnetic resonance imaging, sacroiliitis, juvenile spondyloarthritis disease activity, juvenile arthritis disease activity score

## Abstract

Enthesitis related arthritis (ERA) is a specific subtype of juvenile idiopathic arthritis (JIA), often regarded as an undifferentiated form of juvenile spondyloarthritis (jSpA). While gut is increasingly recognized as origin and/or target of inflammation in adult onset spondyloarthritis (SpA), the incidence of gut involvement in ERA patients is largely unknown. The aim of this study was to measure the concentration of fecal calprotectin (fCAL), a surrogate marker of gut inflammation, in patients with different subtypes of JIA, as well as to correlate the results with various demographic, clinical, laboratory, imaging, and treatment characteristics. The cross-sectional exploratory study involving 71 patients with ERA, other forms of JIA and children complaining musculoskeletal symptoms was therefore conducted. Along with fCAL assessment, a detailed clinical and laboratory examination was performed, including the calculation of a composite disease activity scores. Moreover, MRI of the sacroiliac joints was performed in all ERA and other patients complaining of low back pain. The median concentration of fCAL was highest in ERA patients (33.2 mg/kg, *p* = 0.02), with a significant difference between those with inactive and active disease (20.0 vs. 57.4, *p* = 0.01), as well as those with and without MRI signs of sacroiliitis (22.6 vs. 54.3, *p* = 0.04). The fCAL did not differ depending on the NSAID use (23 vs. 20, *p* = 0.18), although weak correlation was observed with the treatment duration (*r* = 0.25, *p* = 0.03). In conclusion, our findings indicate that a parallel inflammation in musculoskeletal system and gut can occur not just in adults with SpA, but in children with ERA as well.

## Introduction

During the past decades, several studies have shown that a substantial number of children with juvenile idiopathic arthritis (JIA) have some kind of gastrointestinal (GI) symptoms, while up to 85% of JIA patients with significant GI symptoms have histological evidence of mild non-specific inflammation (1–5). Although it has been reported that JIA patient might experience abdominal pain related to NSAID use, other causes should be suspected as well, most importantly gut inflammation (6–9).

Among different subtypes of JIA, gut inflammation is most commonly associated with enthesitis related arthritis (ERA), often regarded as undifferentiated form of juvenile spondyloarthritis (jSpA) (10–12). Despite some differences between spondyloarthritis (SpA) in children and adults, mostly in tendency to involve axial joints which is more remarkable in adults, there are emerging views that spondyloarthritis (SpA) surpasses this arbitrary age-based divide (13). Nevertheless, it is still unclear if clinically silent macroscopic and microscopic gut inflammation which occurs in about 60% of adult patient with ankylosing spondylitis (AS) is present in children with ERA as well (14). This is largely due to the challenges imposed by the use of endoscopy, the gold standard for detailed assessment of the inflammation in the gut, which are considerably important in children (15). Since this procedure is invasive, has to be performed under general anesthesia, and has a potential for rare procedural accidents, such as bleeding and perforation, many children, and their parents experience discomfort, anxiety, and dissatisfaction (16, 17). Besides, symptoms of gut inflammation, such as abdominal pain and diarrhea, are rather vague, particularly in children, and overlap with symptoms of functional gastrointestinal disorders, which makes distinguishing those two rather challenging (18). Therefore, non-invasive tests such as blood and fecal biomarkers are increasingly used in clinical practice to help select patients who might benefit from endoscopies and other more detailed investigations.

In recent years, fecal calprotectin (fCAL), a member of the S100 calcium-binding protein family expressed in phagocytes, monocytes, macrophages and granulocytes, has emerged as a valuable screening tool for the gut inflammation, both in adults and children (19). Moreover, it has been shown that fCAL correlates with endoscopic and histologic inflammatory activity (20). Although many studies have shown increased fCAL in adult SpA patients, there are only few studies of fCAL concentration in JIA and/or jSpA patients (21–25). Furthermore, to best of our knowledge, no study investigated the possible association of fCAL concentration in JIA patients with disease activity indices, presence of sacroiliitis, treatment modalities, and various demographic data, which are all characteristics associated with microscopic gut inflammation in adult SpA patients (26–31).

The aim of this study was therefore to assess the fCAL concentration in patients with various subtypes of JIA and children complaining musculoskeletal symptoms, as well as to assess the correlation with various demographic, clinical, laboratory, imaging, and treatment characteristics.

## Methods

### Study Design and Population

This was a cross-sectional exploratory study in a cohort of patients followed during the year 2019 at the Division of Clinical Immunology and Rheumatology at the Department of Pedaitrics in Sestre Milosrdnice University Hospital Center, Zagreb, Croatia. All of the oligo- and polyarticular JIA and ERA patients fulfilled the ILAR criteria, while in children complaining musculoskeletal symptoms after careful clinical examination, elimination of inflammatory cause of pain, and exclusion of other diseases, only the diagnosis of flat foot was established.

All of the ERA and oligo- and polyarticular JIA patients complained of an abdominal pain for more than seven days, but none had more severe symptoms that interfered with activity, haematochezia, persistent diarrhea, poor growth, prior abnormal studies of the gastrointestinal tract and/or other defined organic cause (e.g., urinary tract infection) (32). Along with fCAL assessment, a detailed clinical and laboratory examination was performed for each patient, including the calculation of a composite juvenile arthritis disease activity score with 27-joint reduced count (JADAS-27) for those diagnosed with oligo- and polyarticular JIA and ERA, as well as juvenile spondyloarthritis disease activity (jSpADA) for those diagnosed with ERA (33, 34). Moreover, as a part of a standard diagnostic procedure, in all ERA patients, as well as in other patients who complained of an inflammatory low back pain, MRI of the sacroiliac joints (SIJ) along with MRI of thoracic and lumbar spine was performed and analyzed by experienced musculoskeletal radiologist according to consensus definitions of components of the Juvenile Arthritis Magnetic Resonance Image Sacroiliac Joint Scoring System (JAMRIS-SIJ) (35, 36). In all ERA and oligo- and polyarticular JIA patients antinuclear antibodies (ANA) and rheumatoid factor (RF) were determined (data not shown). Moreover, in all ERA patients, as well as in some oligo- and polyarticular JIA patients, the presence of HLA-B27 antigen was determined. The disease in ERA and JIA patients was regarded as inactive if jSpADA was ≤0.5 and JADAS-27 ≤1, respectively, according to the previously reported cut-offs (33, 37). Detailed patients characteristics are shown in Tables 1A–D.

**Table 1:** Demographic data **(A)**, disease characteristics **(B)**, laboratory **(C)** and MRI **(D)** findings, and treatment modalities **(E)** of study participants.

		**ERA**	**JIA**	**NIC**
**(A) Demographics**				
***N*** (% female)		26 (46%)	33 (58%)	12 (67%)
**Age** (median yrs, IQR)		12 (7.7–14.5)	11 (7–14)	13 (6.1–14)
**(B) Disease characteristics**				
**Disease duration** (median mo, IQR)		21 (6–54)	18 (6–66)	/
**Active joints** (median N, IQR)		0 (0–2)	0 (0–3.5)	/
**Active enthesis** (median N, IQR)		1 (0–2)	0	/
**Pain** (median VAS, IQR)^a^		3 (0–5)	1 (0–3)	/
**PGA well-being** (median VAS, IQR)^b^		2.5 (0.2–4)	1.5 (0–2.5)	/
**PGA disease activity** (median VAS, IQR)^c^		1.5 (0.37–3)	1 (0–2)	/
**Morning stiffens**^d^ (*N*)		6	7	/
**Clinical sacroiliitis**^e^ (*N*)		5	0	/
**Abnormal back mobility**^f^ (*N*)		4	0	/
**Uveitis**^g^ (*N*)		1	1	/
**Positive family history**^h^ (*N*)		13	14	/
**(C) Disease activity**				
**JADAS-27** (median, IQR)		/	2 (0–4)	/
**JSpADA** (median, IQR)		1 (0–3)	/	/
**(D) Laboratory findings**				
HLA-B27^*^ (N of positive/N analyzed)		12/26	1/17	/
**CRP** (median mg/L, IQR)		1.0 (0.3–2.9)	1.3 (0.3–2.6)	0.5 (0.3–1.9)
**ESR** (median mm/3,6 ks, IQR)		5 (4–11)	8 (5–11.5)	4.5 (3.2–12.5)
**(E) MRI findings**				
**MR of SIJ** (*N*)		26	10	0
**Inflammation components**^i^	**Bone marrow edema** (*N*)	14	0	0
	**Joint space inflamation** (*N*)	9	0	0
	**Capsulitis** (*N*)	0	0	0
	**Enthesitis** (*N*)	2	0	0
**Structural components**^i^	**Sclerosis** (*N*)	4	0	0
	**Erosions** (*N*)	1	0	0
	**Fatty lesion** (*N*)	0	0	0
	**Ankylosis** (*N*)	0	0	0
**(F) Treatment modalities**				
**NSAID**	***N***	14	25	4
	**Duration** (median mo, IQR)	1 (1–15)	4 (1–21)	1 (1–2)
**cDMARD**	***N***	2	5	0
	**Duration** (median mo, IQR)	9 (3–15)	15 (6–46.5)	/
**bDMARD**	***N***	1	4	0
	**Duration** (median mo, IQR)	3	11 (2.5–17.2)	/

*ErA, enthesitis related arthritis; JIA, oligo- and polyarticular juvenile idiopathic arthritis; NIC, non-inflammatory controls; NSAID, non-steroidal anti-inflammatory drug; cDMARD, conventional disease modifying antirheumatic drug; bDMARD, biological disease modifying antirheumatic drug*.

a *patient reported pain over the past week, recorded on a visual analog scale (0, 10)*.

b *patient/parent's global assessment of a child's overall well-being in range from 0 (best) to 10 (worst)*.

c *physician global assessment of disease activity in range from 0 (best) to 10 (worst)*.

d *morning stiffness for >15 min*.

e *defined as the presence of 2 or more of the following: tenderness on examination, positive Patrick's or FABER test and inflammatory back pain*.

f *abnormal back mobility defined as modified Schober's <20 cm*.

g *presence of any uveitis (including acute/symptomatic and chronic/asymptomatic disease)*.

h *ankylosing spondylitis, enthesitis related arthritis, sacroiliitis with inflammatory bowel disease, Reiter's syndrome, or acute anterior uveitis, or a history of one of these disorders in a 1 relative*.

i *as defined by Consensus definitions of components of the Juvenile Arthritis Magnetic Resonance Image Sacroiliac Joint Scoring System (JAMRIS-SIJ) (36)*.

* *the presence of HLA-B27 was not determined in all of the patients*.

### Ethics Statement

The study was conducted per the Helsinki Declaration with the approval from Ethics Committee of the Sestre Milosrdnice University Hospital Center (1-2019-EP). The data were anonymous, and informed consent was obtained from parents/legal guardians as well as from children older than 12 years who participated in the study. All experiments were performed in accordance with relevant guidelines and regulations.

### Fecal Calprotectin (fCAL)

Fecal calprotectin was measured by PETIA (particle enhanced turbidimetric immunoassay) on automatic biochemistry analyzer Architect c8000 (Abbott Laboratories, Illinois, USA) using Bühlmann fCAL® turbo assay (BÜHLMANN Laboratories AG, Schönenbuch, Switzerland). Fecal samples were extracted with extraction buffer using the CALEX® Cap extraction device. The extracts were incubated with reaction buffer and mixed with polystyrene nanoparticles coated with calprotectin-specific antibodies. Calprotectin from the sample mediated the immunoparticle agglutination. Sample turbidity caused by calprotectin-immunoparticle complex formation was proportional to calprotectin concentration. The lower and upper limits of the detection were 20–8,000 mg/kg, respectively. According to the manufacturer's instructions, the fCAL levels below 50 mg/kg were considered normal, those between 50 and 200 mg/kg as slightly elevated, and those above 200 mg/kg as elevated, both in adults and children between 4 and 17 years (38).

### Statistical Analysis

Data were analyzed using GraphPad Prism 8 and *p* < 0.05 was considered statistically significant. Normality of distribution was tested with Saphiro-Wilk test and data are presented as interquartile ranges (IQR) and medians. Statistical comparisons between two groups were performed using the Student's *t*-test (normal distribution) or the Mann-Whitney *U* test, while the comparisons between three or more groups were performed by Kruskall-Wallis test, following a *post-hoc* test using Tukey's method. Correlations between the parameters were calculated using the Spearman rank correlation. For the calprotectin test, the results below the detection limits were equalized to 20 mg/kg.

## Results

In total, 71 patients were enrolled in the study. Among them, 26 patients were diagnosed with ERA and 33 patients were diagnosed with oligo- and polyarticular forms of JIA, while the rest were 12 children complaining musculoskeletal symptoms, regarded as non-inflammatory controls (NIC).

### Fecal Calprotectin in Patients With Various Forms of JIA

**Figure 1:**
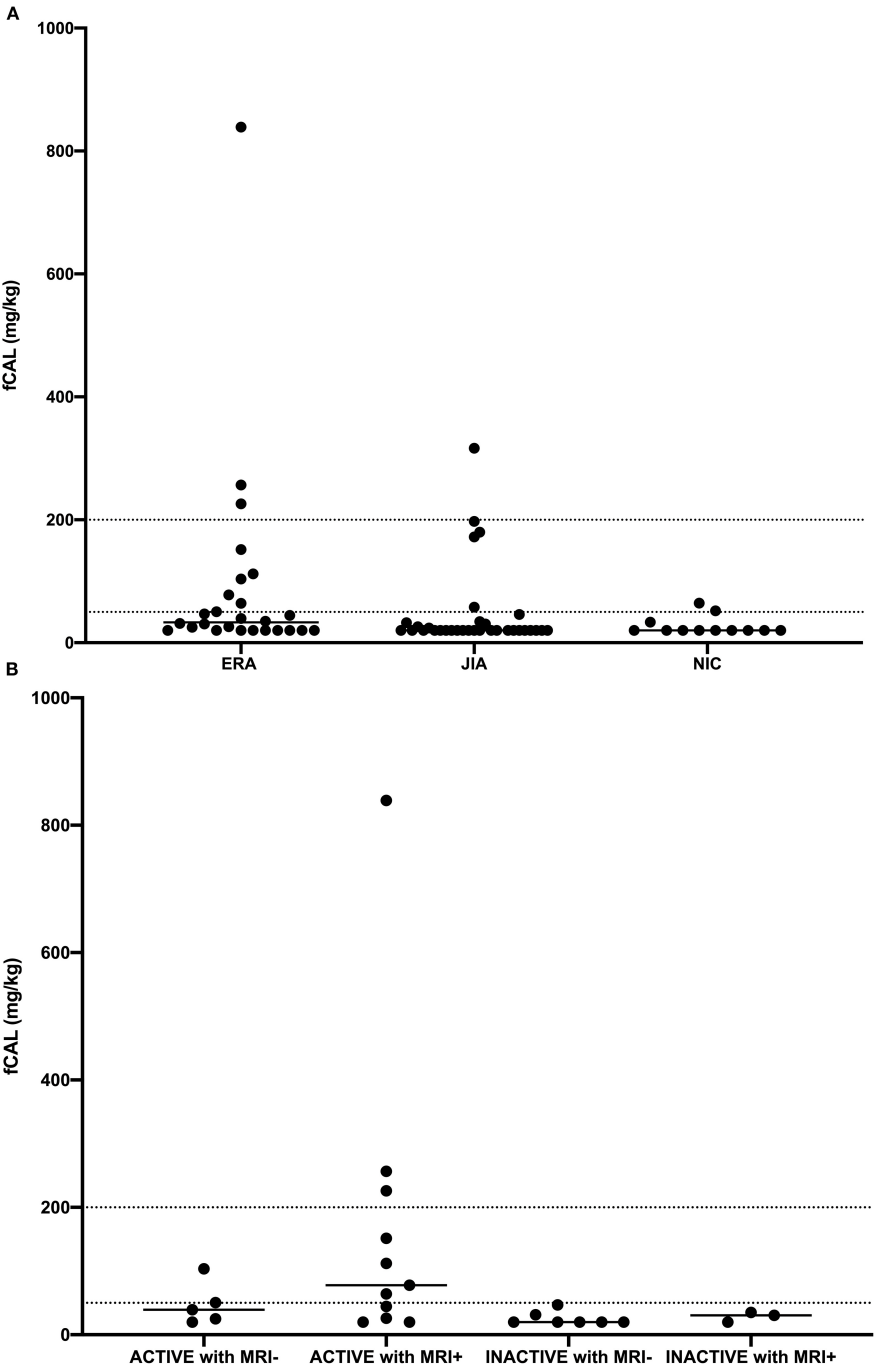
Fecal calprotectin (fCAL) concentration in **(A)** enthesitis related arthritis (ERA), oligo- and polyarticular juvenile idiopathic arthritis (JIA), and non-inflammatory control (NIC) patients, and **(B)** enthesitis related arthritis (ERA) patients with active and inactive disease, with (MRI+) or without (MRI-) inflammatory changes detected by MRI. Each dot represents fCAL concentration in a single patient, while horizontal lines represent median values.

Overall, the median concentration of fCAL was highest in ERA group [33.2 (20–84.8), *p* = 0.02] (Figure 1A). A *post-hoc* test revealed that ERA patients had significantly higher fCAL than other JIA patients (adjusted *p* = 0.04). Moreover, 30.8% of ERA patients had values above 50 mg/kg, which was regarded as abnormal by test manufacturer (38). This percentage was twice lower in other JIA and NIC patients, 15.1 and 12.1%, respectively.

No significant correlation was observed between fCAL concentration and age at the time of sampling, duration of the disease, number of active joints and/or enthesis, physician global assessment, CRP or ESR concentrations, nor disease activity in JIA patients measured by JADAS-27 (data not shown). There was no difference between the fCAL values in JIA patients with inactive (JADAS-27 ≤1) or active (JADAS-27 ≥1) disease (20.0 vs. 20.0 mg/kg, *p* = 0.934).

### Fecal Calprotectin in Patients With ERA

In patients with ERA, moderate correlation was observed between fCAL concentration and the disease activity measured by jSpADA (*r* = 0.46, *p* = 0.02). Besides, there was a significant difference in fCAL concentration between ERA patients with inactive (jSpADA ≤0.5) or active (jSpADA ≥0.5) disease (20.0 vs. 57.4 mg/kg, *p* = 0.01). Moreover, ERA patients with one or more sign of SIJ inflammation detected by MRI (Table 1E) had a significantly higher fCAL concentration than those without the signs of inflammation (22.6 vs. 54.3 mg/kg, *p* = 0.04). Among all patients with ERA, the median levels of fCAL were highest in those with active disease (jSpADA ≥0.5) and MRI sign(s) of sacroiliitis [77.7 (26–226.1) mg/kg, *p* = 0.04] (Figure 1B), with three patients having a fCAL concentration above 200 mg/kg, and three more in 50–200 mg/kg range.

### Fecal Calprotectin and Various Treatment Modalities

Of 71 patients, 43 (60.5%) were treated with non-steroidal anti-inflammatory drugs (NSAID) at the time of sampling for the average duration of 1 (1–17) months (Table 1F). Seven patients were receiving conventional disease modifying antirheumatic drugs (cDMARD) for a median duration of 15 (5–21) months. Biological disease modifying antirheumatic drugs (bDMARD) were used in four patients with oligo- and polyarticular JIA for a median duration of 11 [2.5–17.2] months and in one patient with ERA for 3 months. All of them were receiving TNF-alfa inhibitor adalimumab. Finally, three patients were treated with glucocorticoids (GC) at the time of sampling for the median time of 12 (2–27) months. Of those, two had oligo- and polyarticular JIA [median duration of treatment 19.5 (12–27)] and one had ERA (duration of treatment 2 months).

In all patients, the fCAL concentration did not significantly differ among those receiving and not receiving NSAID [the median value was 23 (20–49.6) mg/kg in patients receiving NSAID vs. 20 (20–33.6) mg/kg in patients not receiving NSAID, *p* = 0.18], although weak correlation was found with the duration of use (*r* = 0.25, *p* = 0.03). No correlation was observed between fCAL levels and other treatment modalities and duration. The median value of fCAL concentration in patients receiving DMARD was 32.6 (20–44.4) mg/kg, higher than in patients not receiving these medications in which the median value was 20 mg/kg (20–46.7, *p* = 0.27). Moreover, the median value of fCAL in patients receiving bDMARD was 26.3 mg/kg (20–51.6), while in those who were not receiving bDMARD, the median value was 20 mg/kg (20–46.7, *p* = 0.95). Comparing the patients receiving and not receiving GC, the fCAL median value was the same, 20 mg/kg (20–39.4 vs. 20–46.7, respectively, *p* = 0.66). Finally, patients receiving medications (NSAID, DMARD, and GC) and patients not receiving any medication had the same median fCAL values of 20 mg/kg (20–46.3 vs. 20–46.1, respectively, *p* = 0.64).

## Discussion

The association of epithelial barrier and joint inflammation has become a focus of attention in both basic and clinical research, with a task to understand the immunopathogenic link and the diagnostic utility (39). Interestingly, it has been proposed recently that a key event in the early stages of ankylosing spondylitis appears to be the association with subclinical Crohn's-like colitis (12). Although there is no consistent confirmation for the genetical, immunological and environmental ties between gut and joints, there are several clinical indications suggesting a close link between gut inflammation and SpA in adults, while the results of our study might help to further establish this link in children (12, 14, 40).

In our study, we have shown that among patients with various subtypes of JIA, the fCAL concentrations were highest in those with ERA subtype, which is alongside similar studies in adult SpA patients and two studies in children (21–25). Intriguingly, almost a third of patients with this particular type of JIA had fCAL concentrations above the range regarded as normal by the test manufacturer (38). The novel finding of our study was that fCAL concentration was significantly higher in ERA patients with MRI sign(s) characteristic for the SlJ inflammation, which adds to the growing number of evidences for a clinical association of gut inflammation and axial spondyloarthritis in adult and pediatric patients (40–43). Moreover, to best of our knowledge, our study was the first to correlate the fCAL concentration with somewhat novel disease activity scores, such as JADAS-27 and jSpADA, with the results showing significantly higher fCAL concentration in ERA patients with active disease (33, 34). Ultimately, the highest level of fCAL was observed in group of ERA patients with active disease and MRI sign(s) of sacroiliitis, suggesting that gastrointestinal inflammation might be a part of a wider inflammatory process present in patients with ERA. Therefore, the results of our study could encourage the wider diagnostic approach to ERA patients, which involves measuring of fCAL especially in patients with active disease and/or MRI signs of sacroiliitis, even without the signs of GI involvement, and vice versa, performing an MRI of SIJ in ERA patients with increased levels of fCAL and active disease, even without the presence of lower back pain. Moreover, since recurrent abdominal pain is very common in children, especially those with JIA and/or taking NSAID, it is useful to inquire about the potential inflammatory cause by performing a simple, economic and non-invasive test such as fCAL (1, 32, 44). Finally, our results could further inform the translational research on the ties between gut and joints. For this cause, fCAL should be observed merely as a surrogate marker correlating well with the inflammatory activity in the gut (20). Therefore, the significantly different concentrations of fCAL between children with oligo- and polyarticular JIA, ERA, and NIC, implies the different level of inflammatory activity and not necessarily the presence of the clinical symptoms characteristic for the inflammatory bowel disease. Nevertheless, as suggested by previous studies, this “subclinical inflammation” could lead to a certain degree of dysbiosis and further development of inflammatory rheumatic diseases (45–48).

As opposite to some previous studies, the fCAL levels in our study were not associated with the use of NSAID, nor with the use of other treatment modalities for JIA (25). It has been shown that NSAID use in adults may result in the intestinal inflammation and an increase in fCAL levels, while the drug discontinuation results in the decline of the FC levels, suggesting healing of the gut mucosa (49). Since we performed a cross sectional exploratory study with the focus on real-life data, with all of our JIA and ERA patients experiencing only light abdominal pain, none of the treatment modalities was omitted before the sampling for fCAL measurement. Regardless, the fCAL levels were highest in ERA group in which less proportion of patients (14/26) was treated with NSAID than in oligo- and polyarticular JIA group (25/33).

There are several important limitations to our study. Although the best available evidence in the literature support the use of fCAL in inflammatory bowel disease (IBD) diagnosis and monitoring, as well as in distinguishing between IBD and irritable bowel syndrome, the gold standard for the detection of (sub)clinical gut inflammation remains the endoscopy and biopsy, which was in our study performed only in one patient (data not shown) (50, 51). Moreover, we didn't report on repeated values of the fCAL measurement. Nevertheless, this might be of lesser importance, since the primary aim of our study was to assess the presence of the gut inflammation in patients with various forms of JIA via the use of surrogate non-invasive biomarker in a prospective cohort of consecutive patients followed at a single pediatric rheumatology center, regardless of their clinical characteristics, disease status and/or concomitant therapy. Additionally, although sensitive for mucosal inflammation, fCAL is non-specific and environmental exposures such as low fiber intake, lack of physical exercise and increasing age, and/or use of certain drugs such as proton pump inhibitors, can cause its elevation (52–54). Nevertheless, since children in our cohort were of similar age (Table 1A) and from same geographic area, it is reasonable to assume they also had a comparable nutritional habits and physical activity levels, while none of them was taking drugs such as proton pump inhibitors. Finally, the number of the study participants was not high, thereby limiting power of the analysis.

In conclusion, the results of our study show that ERA patients have significantly higher fCAL levels than those with other form of JIA or children complaining musculoskeletal symptoms. Moreover, the concentration was highest in ERA patients with active disease and the MRI sign(s) of the inflammatory process in SIJ, which emphasizes that a parallel inflammation in musculoskeletal system and gut can occur not just in adults with SpA, but also in children with undifferentiated SpA. Although this observation still needs to be confirmed in a multicentric studies involving larger number of patients, it could already contribute to planning of diagnostic procedures and treatment modalities in the clinical care for ERA patients, but also further encourage the translational research of the link between gut and joints.

## Data Availability Statement

The original contributions presented in the study are included in the article/supplementary material, further inquiries can be directed to the corresponding author.

## Ethics Statement

The studies involving human participants were reviewed and approved by Ethics Committee of the Sestre Milosrdnice University Hospital Center. Informed consent was obtained from the participant's legal guardian/next of kin, as well as from participants older than 12 years.

## Author Contributions

LL: study design, charts review, data interpretation, statistical analysis, and manuscript preparation. MM: data interpretation, statistical analysis, and final review of the manuscript. RV and MV: data interpretation and final review of the manuscript. ML and IT: charts review and manuscript preparation. NG: laboratory analysis, statistical analysis, and final review of the manuscript. MH: final review of the manuscript. All authors contributed to the article and approved the submitted version.

## Conflict of Interest

The authors declare that the research was conducted in the absence of any commercial or financial relationships that could be construed as a potential conflict of interest.
